# Meeting the unique challenges of drug discovery for neurodegenerative diseases

**DOI:** 10.1186/1471-2377-9-S1-I1

**Published:** 2009-06-12

**Authors:** Diana W Shineman, Howard M Fillit

**Affiliations:** 1 Alzheimer's Drug Discovery Foundation, 1414 Avenue of Americas Suite 1502, New York, NY 10019, USA

## Introduction

There is a mounting need for therapeutics to effectively treat neurodegenerative diseases. Alzheimer's disease, Parkinson's disease, Huntington's disease, amyotrophic lateral sclerosis, and multiple sclerosis almost all share pathological hallmarks of accumulated misfolded protein, ultimately leading to cellular degeneration and death [1]. There is much to be learned by the successes and failures of drug discovery efforts for these respective diseases. Exciting and novel ideas from academia often fail to reach drug discovery platforms and pharmaceutical companies have had little success in their neurodegenerative disease programs thus far; currently, only symptomatic treatments are available for the majority of these diseases [2,3].

While these diseases present unique challenges in terms of drug discovery, they also offer many opportunities to change the way academics and industry work together to efficiently develop new drugs. To bring new drugs into clinical practice for neurodegenerative diseases, efforts to translate academic discoveries into drug discovery and development efforts can be expanded and partnering between academic biologists, medicinal chemists and industry researchers encouraged. Cross-fertilization of ideas between these different neurodegenerative diseases as well as between academia and industry will foster novel developments and hopefully bring us closer to developing effective treatments for these diseases.

These proceedings highlight new approaches to address and overcome the specific challenges of drug discovery for neurodegenerative diseases that were discussed at the 3rd Drug Discovery for Neurodegenerative Conference (held in Washington DC on 2–3 February 2009). This conference was hosted by the Alzheimer's Drug Discovery Foundation, in partnership with the National Institutes of Health, to advance drug discovery for neurodegenerative diseases by educating scientists on the process of translating basic research into novel therapies. Over the two day conference, speakers presented lectures and case studies on Alzheimer's disease, Parkinson's disease, Huntington's disease, amyotrophic lateral sclerosis, multiple sclerosis, as well as orphan neurological diseases. All of these diseases share common challenges and require a broad and coordinated, multi-disciplinary approach to progress novel discoveries into effective therapeutics.

## Basics of medicinal chemistry

Medicinal chemistry is an essential piece to the drug discovery puzzle and was highlighted in the first session of the conference. This session was chaired by Dr Martin Watterson (Northwestern University) and included talks on the fundamentals of drug discovery chemistry and how later-stage considerations of pharmacokinetics, pathophysiology and production must be considered early on, at the medicinal chemistry stage. Dr Alan Kozikowski (University of Illinois) reminded participants that intuition plays an important role in medicinal chemistry as well and biologists need to work in partnership with medicinal chemists in a coordinated effort. The importance of patents and intellectual property in medicinal chemistry was also discussed. Rick Silverman (Northwestern University) discussed a case study of developing selective neuronal nitric oxide synthase inhibitors [4]. Drs Frank Longo (Stanford University) and Jordan Tang (Oklahoma Medical Research Foundation) also presented fascinating case studies about developing drugs to target key pathways in neurodegenerative diseases. Frank Longo discussed virtual library screening and his experience developing peptidomimetic compounds [5], while Jordan Tang used his expertise on matrix metalloproteases to develop novel and specific inhibitors of beta-secretase [6]. Finally, Dr Chris Lipinski (Melior Discovery) closed the session with a discussion on selection of candidates for drug development, emphasizing that his 'Lipinski Rule of 5' criteria should be used as guidelines and not as steadfast rules.

## Hits and leads: early phases of drug discovery

Marcie Glicksman (Harvard Medical School) discussed common pitfalls in high-throughput development and emphasized the importance of developing high quality secondary screens. This point was also emphasized by Dr Linda VanEldik (Northwestern University), who discussed the utility of also performing secondary screens to determine ADMET (absorption, distribution, metabolism and excretion, and toxicology) properties and noted that many of these screens are commercially available. Most drug development efforts fail due to toxicity and ADMET properties and Dr Karen Steinmetz (SRI International) went into more detail on some of the technologies for ADMET screening, discussed common pitfalls and emphasized the need to consider the future plan of clinical administration at the onset of screening. An in-depth review of ADMET considerations in drug discovery is presented in these proceedings by Katya Tsaioun (Apredica).

## Pre-clinical proof-of-concept and development

Once a drug-candidate has been identified, pre-clinical proof-of-concept and drug development studies are initiated, as described by Edward Spack (SRI International) at the conference and as presented here in these proceedings. In transitioning from lead compound to clinical testing, William Banks (Saint Louis University) discussed preclinical proof-of-concept and the challenges of the blood-brain barrier in central nervous system drug discovery, as well as methods to improve central nervous system exposure. A review of characteristics of compounds that cross the blood-brain barrier is presented by Dr Banks in these proceedings. In addition, Nancy Wehner (Elan Pharmaceuticals) discussed the requirements that lead compounds should meet before becoming 'clinical candidates' and emphasized that outsourcing pre-clinical studies is often needed to efficiently move a compound forward. Daniela Brunner (PsychoGenics, Inc.) discussed behavioral testing in neurodegenerative diseases from a drug screening perspective [7]. Finally, partnership opportunities are often crucial to moving clinical candidates forward into human testing. Thomas Argentieri (Wyeth) addressed what decisions may support or hinder pharma partnership opportunities. He stressed the importance of intellectual property position and discussed the major attributes companies look for in a partner.

## Issues in technology transfer: interactions and intellectual property

The importance of protecting intellectual property position was echoed in a session chaired by Kathleen Denis (Rockefeller University). This session began with an introduction on the various roles and responsibilities of the tech transfer office as well as the basics of patents and what they can and cannot successfully cover. The requirements for patentability were further outlined by Colin Sandercock (Proskauer Rose LLP). Louis Berneman (Texelerate) then went on to discuss patent licensing and material transfer agreements as ways for academics to create and foster relationships with industry that are beneficial to both parties. Finally, John Swartley (University of Pennsylvania) closed the session with an assessment of the positives and negatives of starting a biotechnology company, stressing the importance of managing expectations and setting realistic company goals.

## Resources and services for advancing drug discovery

The final session of the conference focused on resources and services available for advancing drug discovery and included talks on the scientific and funding resources available. These mechanisms include those within academia (as presented by Martin Watterson), within the National Institutes of Health (as presented by Suzana Petanceska and Lorenzo Refolo), through disease-specific foundation resources, and through commercial vendors (as presented by Katya Tsaioun, Apredica). Speakers focused on resources for assay development, target identification, drug discovery, drug development, pre-clinical toxicology evaluation and other components needed for the translation of pre-clinical drug candidates into potential therapies tested in clinical trials. The final panel discussed the role of venture philanthropy in funding drug discovery for neurodegenerative disorders. Philanthropies are able to take on more risk and fund novel high risk approaches that would be less likely to be funded through more traditional mechanisms.

## Conclusion

This conference provided ample networking opportunities for scientists from academia, biotechnology companies, and contract research organizations to meet, discuss ideas and foster collaborations. Through continued education and the nurturing of interdisciplinary interactions, we will be able to overcome the unique challenges of drug discovery for neurodegenerative disease and develop effective therapies to meet the growing need for treatments.

## List of abbreviations used

ADMET: absorption, distribution, metabolism and excretion, and toxicology.

## Competing interests

The authors declare that they have no competing interests.
